# Inflicted head-injury by shaking-trauma in infants: the importance of spatiotemporal variations of the head’s rotation center

**DOI:** 10.1038/s41598-023-42373-x

**Published:** 2023-09-14

**Authors:** L. A. H. Schiks, J. Dankelman, A. J. Loeve

**Affiliations:** 1 Department of Biomechanical Engineering, Faculty of Mechanical, Maritime and Materials Engineering, Delft University of Technology, Delft, The Netherlands; 2 Co van Ledden Hulsebosch Center for Forensic Science and Medicine, Amsterdam, The Netherlands

**Keywords:** Paediatrics, Biomedical engineering

## Abstract

Inflicted head injury by shaking trauma (IHI-ST) in infants is a type of abusive head trauma often simulated computationally to investigate causalities between violent shaking and injury. This is commonly done with the head’s rotation center kept fixed over time. However, due to the flexibility of the infant’s neck and the external shaking motion imposed by the perpetrator it is unlikely that the rotation center is static. Using a test-dummy, shaken by volunteers, we demonstrated experimentally that the location of the head’s rotation center moves considerably over time. We further showed that implementation of a spatiotemporal-varying rotation center in an improved kinematic model resulted in strongly improved replication of shaking compared to existing methods. Hence, we stress that the validity of current infant shaking injury risk assessments and the injury thresholds on which these assessments are based, both often used in court cases, should be re-evaluated.

## Introduction

Inflicted head injuries by shaking are diagnosed in 14–41 per 100.000 young children annually. The highest incidence is in the early infant ages, similar to abusive head trauma in general^1–3^. Retinal hemorrhage, subdural hemorrhage, diffuse axonal injury, and neck injury are often associated with violent shaking. The diagnosis of inflicted head injury by shaking trauma (IHI-ST) based on these symptoms is often debated, as these can also be caused by other events, such as traffic accidents or maternal, obstetric and neonatal factors^4–7^. Furthermore, there is no consensus yet about whether shaking alone can result in loading and deforming an infant’s anatomical structures beyond their failure thresholds and cause the abovementioned injuries^8–11^. This makes legal cases concerning potentially maltreated or abused infants complex and often inconclusive, and may potentially have caused mistrials in an unknown number of cases, either letting infants stay with abusive caretakers or taking them away from innocent parents.

Zandwijk et al.^12^ found that a reason for the lack of consensus may be the seemingly conflicting results obtained from different shaking injury assessment studies. Gross head accelerations and velocities measured for abusive shaking were concluded by several experimental studies to be too low to surpass the commonly used injury thresholds for such kinematic parameters^13–16^. Yet, various other experiments^17–19^ and computational studies^20–26^ using detailed models of the anatomical structures inside an infant’s head generally conclude that the same shaking kinematics cause loading and deformation of brain, eye and bridging vein tissues that do exceed their ultimate strengths.

Besides the fact that infant shaking trauma assessments often use injury thresholds that are based on extrapolated or scaled adult- or animal data or that are not based on shaking at all (as shown by Schiks et al.^27^), a potentially crucial aspect of the head motion during shaking seems to be commonly neglected. Measurements of gross head motion are often aimed at a limited set of kinematic parameters, such as angular velocity and acceleration of the top of the skull^12^, which only partly describes the head motion. Computational studies on IHI-ST, such as finite element models (FEM) and rigid body models (RBM) of an infant’s anatomy are commonly subjected to dynamics that have been measured in physical model studies^10,13–15,19,28–31^. Where in RBMs the load applied to the chest is often a simple translation or linear acceleration^31–34^, loads applied to FEMs are often more complex. The head may be subjected to a uniaxial sinusoidal displacement^35–37^, or to—whether or not combined—linear or angular accelerations or velocities^20–22,38–40^. The center of rotation of an infant’s head in such FEM simulations is usually defined at a fixed point somewhere at the neck; e.g. the base of the skull, the C5-C6 junction or the base of the neck^20–22,38–40^. Consequently, physical shaking and computational shaking simulation studies generally seem to disregard—in their calculations and in applying measured kinematics to computational models—that the instantaneous center of rotation (ICOR) of a child’s head can be expected to fluctuate during shaking.

The instantaneous radius of curvature (IROC)—the distance between the head’s center of gravity (COG) and its ICOR—determines the nature of the relative motion between the skull and, e.g. the brain during motion, and hence the loads and deformations of the brain^20,41–43^. A small IROC predominantly causes rotation of the brain with respect to the skull, whereas a larger IROC results in more linear displacement of the brain within the skull^20,44^. Rotational loads are associated with ruptured bridging veins and diffuse injuries, while translational loads are more associated with contusions and focal injuries^41,45,46^. Hence, proper measurement of head kinematics, including its ICOR, and applying these in computational studies is crucial to assess, for example, whether a child could have fallen off a chair accidentally or must have been shaken abusively.

It is currently unknown how the ICOR of a child’s head varies spatiotemporally during shaking and how this manifests itself in the expression of injury mechanisms associated with IHI-ST. Therefore, it is unknown to what extent existing IHI-ST computational studies accurately replicate the tissue-loading that results from shaking, or even how the kinematic results of most existing experimental studies should be interpreted.

The aims of this study were:

1. To determine the spatiotemporal variation of the ICOR of a surrogate infant’s head during shaking in order to discuss its potential effect on injury mechanisms related to IHI-ST.
2. To assess to what extent existing studies using fixed centers of rotation could be improved by using spatiotemporally varying centers of rotation.

**Figure 1:**
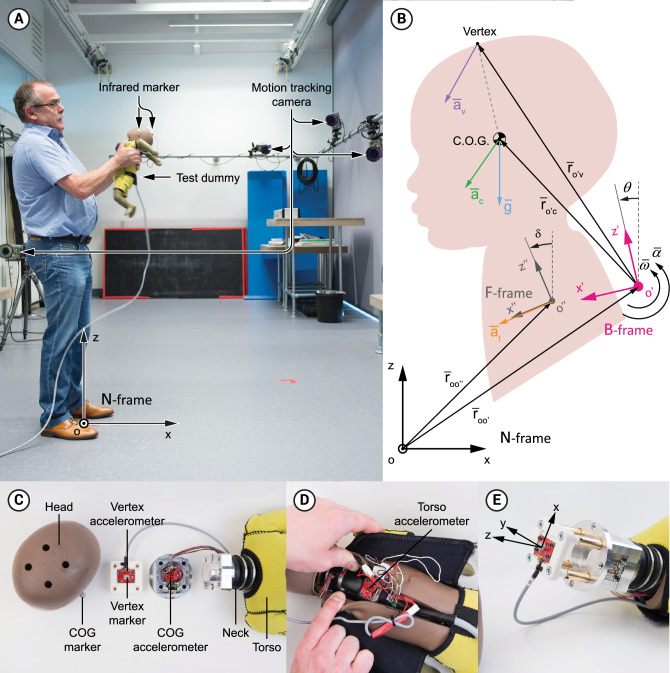
Experimental setup. (**A**) Overview of the camera set-up (with permission of the participant). (**B**) Kinematic diagram of the infant’s head and torso. Inertial reference frame N is defined by the xyz triad. Its origin corresponds with the marking on the floor at the participant’s feet; the same place at which the dummy’s sensors and the motion tracking system were zeroed at. Moving reference frame B rotates with the head and is defined by the x’y’z’ triad according to the anatomical definition (x-axis dummy face forwards, y-axis to the dummy right, z-axis upwards). The B-frame is tilted by angle θ with respect to the z-axis of the inertial reference frame N, and its origin o’ corresponds with the ICOR—both tilt angle θ and origin o’ vary over each timeframe. Torso reference frame F rotates with the torso and is defined by the x”y”z” triad according to the anatomical definition, with its origin at the torso accelerometer. The F-frame is tilted with respect to the z-axis by angle δ, which varies over each timeframe. Other variables are further explained in Eqs. (1) to (5) in the “Methods” section. (**C**) Components of the dummy’s head. (**D**) Position of the torso accelerometer. (**E**) Sensing axes according to a right-hand coordinate system with respect to human body axes: x-direction, longitudinal axis, anterior positive; y-direction, transverse axis, left positive; z-direction, vertical axis, superior positive. Flexion of the neck represents a positive rotation around the y-axis. The participant shown in the figure gave informed consent for being depicted.

We investigated the role of the ICOR in IHI-ST modeling in a two-part study. First, a surrogate study was conducted in which 33 volunteers fiercely shook an instrumented test-dummy (mimicking a 6 weeks-old infant, see Fig. 1) to allow measuring the dummy’s head and torso kinematics during shaking. Next, a computational study was conducted to compare the modelling accuracies of existing shaking kinematics models obtained from literature (which use fixed centers of rotation for the head) with a newly proposed model (which uses a moving center of rotation for the head).

## Results

**Figure 2:**
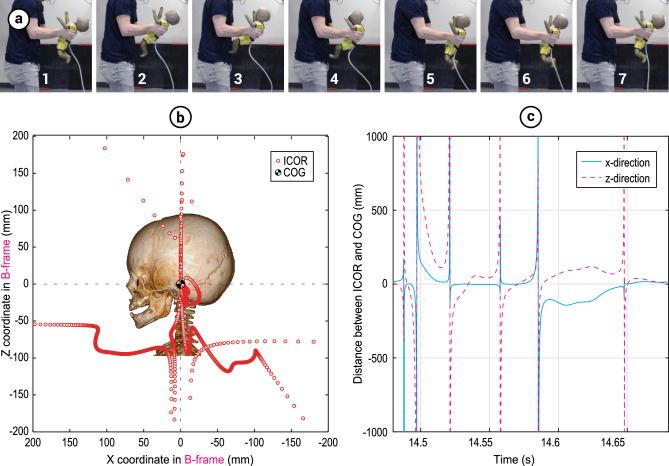
Visualization of shake cycle characteristics. (**a**) Subsequent stages in the full shake cycle of the dummy (**1**–**7**). The shake cycle starts (**1**) when the dummy neck is fully extended, half way the cycle (**4**) the dummy neck is fully flexed and at the end of the cycle (**7**) the situation is equal to (**1**) again. (**b**) Graphical representation of spatiotemporal variation of the instantaneous center of rotation (ICOR) with respect to the center of gravity (COG) of the head, during a single typical shake cycle, mapped on a 3D CT-scan of a 6-week old infant for illustrative purposes. (**c**) Typical example of the location of the instantaneous center of rotation (ICOR) over time during a single shake cycle with respect to the center of gravity (COG) of the dummy’s head.

Motion capture and accelerometer data were acquired while having the test-dummy shaken vigorously by 29 participants (mean age 33 years, range 21 to 64, 8 female and 21 male, 4 of the originally 33 participants were excluded due to dummy failure). These showed that the location of the dummy’s head’s ICOR varied largely over time within each shake cycle, in both x- and z-direction with respect to the COG (Fig. 2). There were no significant correlations between the participants’ age, weight or height, and the obtained kinematics (Supplementary Table S4).

**Figure 3:**
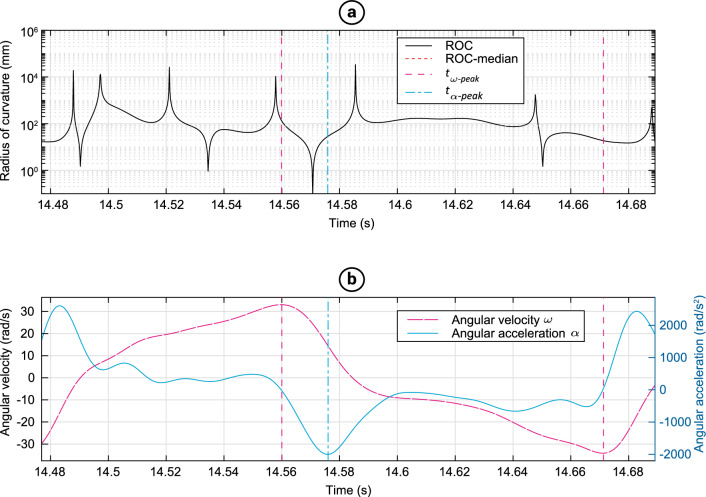
Temporal relation between peak values in a single shake cycle of participant 16. (**a**) Instantaneous radius of curvature (IROC) of the head’s center of gravity during a single typical shake cycle. The instances at which the peak angular velocity and acceleration occur are indicated by vertical lines t_ω-peak_ and t_α-peak_ respectively. (**b**) angular velocity of the head and angular acceleration of the head during a single typical shake cycle. The IROC was highly variable at some points in the shake cycle, also in the proximity of angular acceleration peaks.

**Figure 4:**
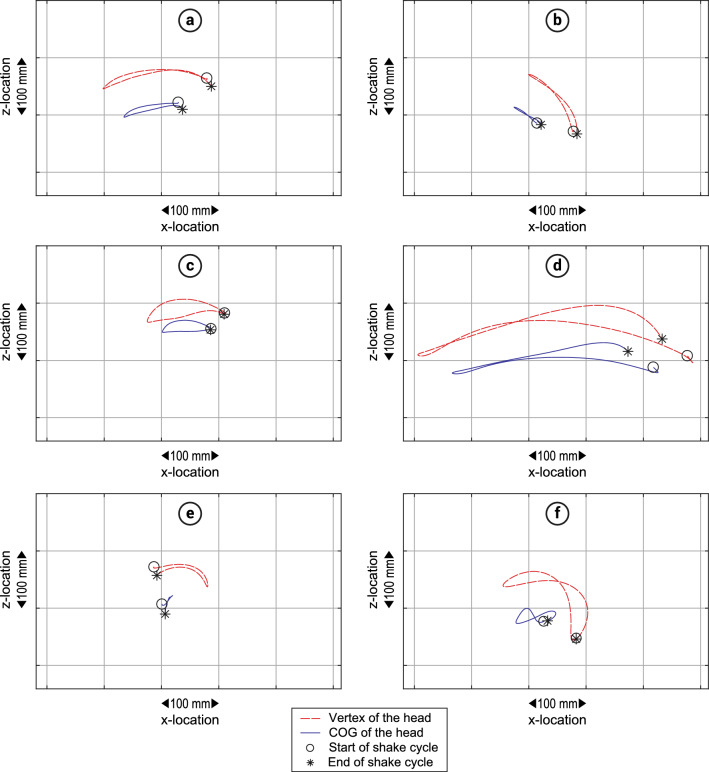
Typical examples of shaking pattern variations encountered during the study. (**a**,**d**) Small vs. large amplitude. (**d**,**f**) Weakly curved vs. strongly curved. (**c**,**f**) Circular vs. eight-shaped. (**a**,**e**) Little vs. much vertical displacement of the COG. (**a**,**b**) Horizontal vs. gravity assisted shaking. Coordinates are expressed in the inertial reference frame.

The mean value, across all participants, of the median IROC during the shake cycle with the highest vertex acceleration was 96 mm, SD = 41 mm. The IROC fluctuated largely in the proximity of angular acceleration peaks (Fig. 3). The minimum and maximum IROC observed were 3.1E-6 m (considered pure rotation) and 1.7E3 m (considered pure translation) respectively. The IROC at the instance of the absolute maximum ω and α had a median over all participants of 29 mm (min. 2 mm, max. 244 mm) and 38 mm (min. 3 mm, max. 156 mm) respectively. Among the participants there were considerable variations in the trajectories of the dummy’s head during shaking. Notable differences in the trajectories were found in amplitude, curvedness, shape (circular vs. eight-shaped), amount of vertical displacement of the COG, and shaking direction (horizontal vs. gravity assisted, i.e. shaking the infant in a more downward direction to make use of the gravitational acceleration) (Fig. 4). The peak values of the measured kinematic variables and the average shaking frequency f_shake_ were defined over the entire trial of each participant and are summarized in Supplementary Table S5 (See “4TU Centre for Research Data” repository for full dataset^47^).

The fixed-ICOR models identified in the literature were categorized as:

- CAT (I) Only rotation of the head, with the fixed rotation center—either the base of the neck^20^ or the C5-C6 junction^21,38^—aligned with the head’s center of gravity. Input kinematics were rotational acceleration and velocity of the head.
  - SubCAT (I-A) Head rotation center at the neck base.
  - SubCAT (I-B) Head rotation center at the C5-C6 junction.
- CAT (II) Only translation of the head, hence with a rotation center at infinity. Input kinematics were linear (horizontal or anterior–posterior) accelerations of the model^35–37^.
- CAT (III) Combined rotation and translation of the head. Input kinematics were the torso linear acceleration vector applied at the chosen rotation center of the head, assuming no torso rotation with respect to the inertial reference frame^22,39,40^.
  - SubCAT (III-A) Head rotation center at the neck base.
  - SubCAT (III-B) Head rotation center at the skull base.

The equations of motion (EOM) for all categories of fixed-ICOR models and the new moving-ICOR model are provided and explained in the “Methods” section. The performance of these models for calculating the head output kinematics, after feeding them all with the same input data obtained from one of the test runs from the conducted shaking experiment, showed large differences, both local and overall.

**Figure 5:**
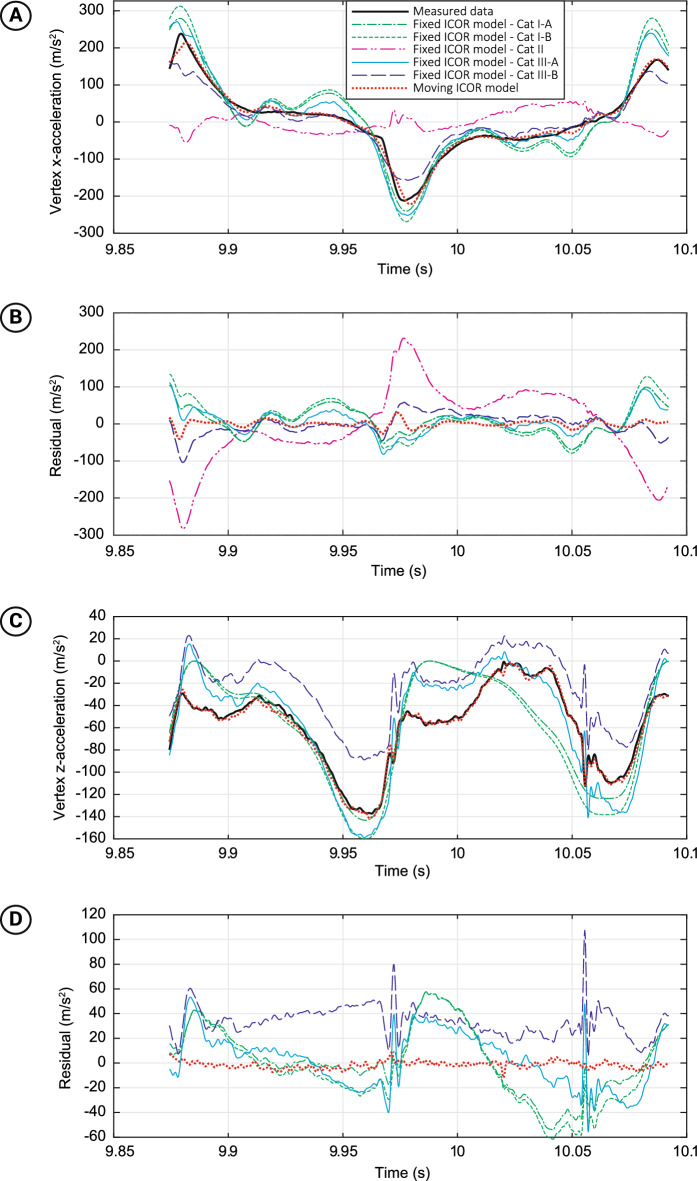
Replication of vertex accelerations of the head in x-direction and z-direction during the single shake cycle in which the highest tangential vertex acceleration was reached by—arbitrarily chosen—participant 1. (**a**) Inertial vertex acceleration in x-direction. (**b**) Residual acceleration in x-direction. (**c**) Inertial vertex acceleration in z-direction. (**d**) Residual acceleration in z-direction. The legend in (**a**) is for all sub-figures.

The moving-ICOR model outperformed all fixed-ICOR models in replicating the shaking kinematics. The mean absolute root-mean-square errors (RMSE) over a full shake cycle are provided in Table 1. The CAT II models, which incorporated horizontal translation only, performed far worse than other model-loading categories. The moving-ICOR model had the smallest RMSE in both x- and z-direction. The acceleration residuals of the moving-ICOR model were smaller and more consistent over the entire shake cycle for x- and z-direction as compared to the fixed-ICOR models categories. Particularly the CAT II methods had large residuals at acceleration peaks in x-direction (Fig. 5a,b). All fixed-ICOR models had large residuals for the acceleration in z-direction (Fig. 5c,d). The results for the COG accelerations were similar to those of the vertex.

**Table 1:** Model performances for replicating shaking kinematics.

	Vertex		COG	
	RMSE-X (m/s^2^)	RMSE-Z (m/s^2^)	RMSE-X (m/s^2^)	RMSE-Z (m/s^2^)
CAT I-A	46.2	39.9	43.3	38.4
CAT I-B	56.3	44.3	53.7	42.6
CAT II	101.1	N/A*	49.2	N/A*
CAT III-A	41.0	38.3	36.8	36.6
CAT III-B	33.6	42.7	30.5	42.6
Moving-ICOR model	12.1	6.3	0.0**	0.0**

Absolute root-mean-square error (RMSE) between the models and the measured accelerations of the head’s vertex and center of gravity (COG) for each participant’s selected shake cycle, averaged across all participants. (*) The RMSE-Z was not calculated for CAT II because this model-type only involved horizontal accelerations. (**) The RMSE-X and -Z of the COG for the Moving-ICOR model are both zero because the kinematic values used as input for the model were directly derived from the measured COG accelerations. Data of each participants’ shake cycle with the highest tangential vertex acceleration was used.

## Discussion

By asking the participants in the current study to shake an instrumented dummy as hard and for as long as possible, we attempted to estimate the limit fierceness of shaking that may be exerted by human participants. Various peak values of the measured kinematic variables (torso-, head COG-, and vertex linear accelerations; head angular velocity and acceleration; and shaking frequency) were well outside the range of mean + /– 1.96 SD. This underlines the importance of realizing that there can be a difference between what an average human and what a specific person (or suspect) is physically capable of.

To the best of the authors’ knowledge, this study is the largest infant surrogate shaking study available, as existing studies had 1 to 11 participants^10,13–15,19,28,29^. Still, legal cases could greatly benefit from knowing what shaking fierceness can be obtained by a person depending on their build and physical condition. This would require studies with many more participants than in the current study. Furthermore, induced anger feelings and encouragement of the participants during shaking may result in more intense shaking, which could help to find the true limits of shaking fierceness of which a person is capable of. When giving participants a free choice of shaking method, other, even more damaging shaking methods may be discovered. Recruiting also teenage participants in addition to the age groups who participated in this study may help to better reflect the perpetrator demographics reported by others^48,49^.

The location of the ICOR of the dummy’s head was shown to continuously move during shaking. Furthermore, the 1E9 order of magnitude variation of the radius of curvature (IROC) shows that the infant’s head motion during shaking fluctuates between nearly pure rotation and pure translation. Differences between linear accelerations of the head’s vertex and COG were large; peak vertex accelerations were often over twice the corresponding COG accelerations. The difference in acceleration between the head’s vertex and the COG is determined by three factors: (1) the angular acceleration magnitude; causing a difference in the tangential acceleration components, (2) the magnitude of the angular velocity; causing a difference in the normal acceleration components and (3) the location of the ICOR; causing a difference in both the tangential and normal acceleration components. Hence, the location of the ICOR contributes not only to the magnitude, but also to the nature of tissue loads and deformations. Those differences in translational accelerations between the vertex and the COG would create the optimal conditions for inducing shear forces in the brain, but this effect is reduced because the brain is suspended in cerebrospinal fluid. Yet, the very same fluid promotes brain rotation with respect to the skull, which increases the stretching of bridging veins, potentially leading to hemorrhages^35,40^. In order to obtain detailed knowledge about the trauma mechanisms involved in violent shaking of infants it is crucial to implement the measured ICOR fluctuations in future computational and surrogate studies.

It should be noted that the objective of this study was to investigate the principle effect of spatiotemporal variations of the rotation center by intra-study-comparison of computational models, and not to approximate biofidelity as closely as possible. Therefore, the addressed modifications of the dummy and sensor bracket—and thereby the affected biofidelity of the dummy—are considered not to alter the validity of the study or the conclusions drawn from it, but may alter the kinematic measurements and the upper and lower limits of the ICOR. Furthermore, it has been shown previously^13,31,33^ that floppier necks result in higher head accelerations. The used dummy had a neck designed for high speed impact situations and was therefore rather stiff for low speeds, compared to real infant necks. Hence, the accelerations measured in our experiments are believed to still be on the conservative side.

At the moments of peak angular velocity and peak angular acceleration—on which several existing injury thresholds are based^10,11,13,50–53^—the median IROCs were found to be 29 mm and 38 mm, respectively. Computational models in the literature, however, usually define the base of the skull, the C5-C6 junction or the base of the neck as a fixed ICOR^20–22,38–40^; corresponding with IROCs of about 27 mm, 67 mm and 82 mm, respectively. The current study showed that the IROC varies from values much smaller to much larger than these commonly used fixed distances. Hence, it is expected that existing injury thresholds based on peak magnitudes have been overestimated in various studies^10,11,13,50–53^ and that tissue deformations simulated by computational models with the ICOR at the base of the skull, the C5-C6 junction or the base of the neck^20–22,38–40^, have been underestimated. Furthermore, in the proximity of the maxima of the angular velocity and angular acceleration the ICOR showed to be very near the COG of the dummy’s head, while angular accelerations simultaneously were close to their maximum. Therefore, it might be that tissue is loaded at its maximum at a moment that is not necessarily the point of maximum angular acceleration/velocity, due to a combination of high, but sub-maximal angular accelerations with minimal IROC. This would make injury thresholds based on maxima of a single kinematic factor likely to underestimate the true risks.

The assessment of the IHI-ST modeling accuracy of existing fixed-ICOR models and the proposed moving-ICOR model further underlined the importance of taking ICOR motion into account. These computational comparisons showed that the simplifications commonly made when calculating IHI-ST shaking kinematics could largely affect the perceived danger of shaking. At first sight, the CAT III models that do combine rotation and acceleration but with a fixed ICOR provide a reasonable replication of head dynamics. Yet, the absolute error is over twice that of the moving-ICOR model. Moreover, due to ignoring the ICOR motion, at some points fixed-ICOR methods predicted positive accelerations, while the actual accelerations were negative. Accurate temporal replication of shaking kinematics is crucial when modeling biomechanics of viscoelastic tissues such as brain tissue, because the tissue response not only depends on loading magnitude, but also on its rate, repetitions and excitation type^27,40,54^. For that, researchers working on such models could, as a starting point, apply the shaking kinematics from the dataset^47^ published with the current report on the infant head in their rigid body or finite element models.

Studies that used fixed-ICOR models to determine shaking kinematics or to determine soft tissue loading and deformations inside the head may need to be redone using the moving-ICOR model for various specific reasons:

- CAT (I) Morison^20^, compared pure translation, pure rotation around the skull center and rotation around the neck base, and reported that bridging veins stretch mainly due to the rotational motion of the brain in the skull. Roth et al.^21^ compared the intracerebral mechanical response for impact and shaking and found that both shaking and impact may result in a subdural hematoma due to rupture of bridging veins. Raul et al.^38^ investigated the effect of benign enlargement of the subarachnoid space on bridging vein stretch during shaking. These studies used the neck base or C5-C6 junction as rotation center and neglected linear accelerations, which would lead to both over- and underestimations throughout most of a shake cycle. Only minor differences were observed between the outcome of CAT I-A and CAT I-B models, as the rotation center locations were similar.
- CAT (II) Brain tissue motion, loading and deformation has been studied by several groups^35–37^ using models with fixed-ICOR and with only translational input motion. However, by leaving out rotational inputs (which was shown in the current study to be a most relevant part of shaking kinematics) effects such as pressure build-up due to centripetal forces, z-accelerations, vertical and rotational displacement of the brain and the accompanying stretching of bridging veins may have been considerably underestimated. This also suggests that the non-substantiated advice of Couper and Albermani^55,56^ that using only anterior–posterior accelerations suffices for modeling the qualitative mechanics of the head in oscillatory motion should not be followed.
- CAT (III) Hans et al.^22^, Couper and Albermani^39^ and Batterbee et al.^40^ all used fixed-ICOR approaches, but with various locations of the ICOR. Additionally, these models neglected torso rotations. Furthermore, Hans et al.^22^ was the only study in which gravity was simulated. In general, the current study showed that the CAT III models provide quite accurate x-direction accelerations, but underestimated accelerations in z-direction, and at some points in the shake cycle even resulted in positive accelerations that should have been negative or vice versa. This may have considerable consequences for simulations regarding intracranial pressure build-up due to blood accumulation and CSF displacements.

In general, it seems that the potentially harmful consequences of shaking an infant may have broadly been underestimated throughout IHI-ST experimental and computational studies due to neglecting the ICOR motion. Future research should investigate the effect of the spatiotemporal variation of the ICOR on the anatomical structures in the skull (e.g. tissue deformation or intracranial pressure). A moving-ICOR model describing the head motion during shaking, such as provided by the present study, should be used for model-inputs in future IHI-ST simulation studies to improve their accuracy. This should preferably be combined with experimentally determined variations of the ICOR.

The presented findings may have considerable implications for any scenario of head injury that involves combined rotation and translation of the head. The approach and results presented in this study are believed to be of particular value for forensic science, legal medicine, law, and sports and vehicle safety studies.

Concluding, our experiments showed that the location of the instantaneous center of rotation (ICOR) of an infant surrogate’s head varied greatly over time during violent shaking. Currently existing IHI-ST computational studies usually define the ICOR as a fixed point in the cervical spine, while the distance of the ICOR with respect to the head may differ nine orders of magnitude during shaking. A computational, quantitative comparison showed that existing fixed-ICOR models far less accurately model the kinematics of shaking infants than our newly proposed moving-ICOR model. The risk of IHI-ST is likely to have been underestimated for the past decades due to these inaccuracies. Prior studies on injury thresholds, injury assessment and kinematics of inflicted head injury by shaking trauma in infants should be reassessed. It should be taken into account that the instantaneous center of rotation moves during the shake cycles.

## Methods

### Experiment protocol and study population

An infant surrogate shaking experiment was performed at the BioMechanical Engineering department of the Delft University of Technology (Delft, The Netherlands). Participants were instructed to shake an instrumented test-dummy back and forth in its sagittal plane as violently and as long as possible. This instruction was given in order to investigate the maximum accelerations occurring during violent shaking. Approval for this experiment was granted by the university’s Human Research Ethics Committee (study number 698). All experiments were performed in accordance with relevant guidelines and regulations. A total of 33 volunteers participated in the experiment under informed consent. Data of the last 4 volunteers were excluded from the analysis because of a mechanical failure in the test-dummy.

### Instrumented infant surrogate

Motion of the dummy’s head and kinematics during shaking were measured simultaneously by means of sensors in and on the infant surrogate and a motion capture system; both were calibrated prior to and synchronized after the experiments. An instrumented Q0 crash-test dummy (First Technology Safety Systems, Delft, The Netherlands) was used as an infant surrogate (mimicking a 6 week-old, 3.4 kg infant, based on^57^) to capture kinematic data during the shaking experiments; i.e. angular velocity of the head, and linear accelerations in three directions at the torso, at the center of gravity (COG) and at the vertex of the dummy’s head.

A custom-made sensor bracket replaced the original bracket (Fig. 1C to E and Fig. S1) to hold two tri-axial accelerometers—ADXL377, measurement range ± 200 g, Analog Devices, Inc., Norwood Massachusetts, USA—for measuring at the dummy’s vertex and COG of the head. An identical accelerometer was placed in the torso (Fig. 1D).

The sensor bracket was specifically designed to match the dimensions and inertial properties of the original bracket to not compromise the biofidelity of the dummy’s head. However, due to the use of extra sensors and associated mountings the weight of the new bracket was inevitably higher than the original load cell (273 g and 194 g respectively). Corresponding to an increase of 6.7% of the total head weight (1176 g). The location of the center of gravity of the new bracket in x- and y- direction was equal to that of the original load cell. In the z-direction, the center of gravity of the new bracket was slightly shifted due to the new bracket design; 4.4 mm towards the vertex compared to the original load cell (Fig. S1).

A power spectral density analysis of the acceleration data of similar experiments^58^ with the Q0 dummy revealed that the accelerometer signal power beyond 250 Hz was less than – 25 dB. Therefore, it was decided to set the bandwidth of the accelerometers to 500 Hz, providing a safe margin.

### Motion capture system

An Oqus 700 motion capture system (Qualysis, Göteborg, Sweden) was used to record the three-dimensional trajectory of the dummy’s head during shaking. This system consists of 12 motion-tracking infrared cameras, tracking passive 7 mm spherical reflective markers that were attached to the vertex and to the left side of the dummy’s head, coinciding with the z- and y-axis of the dummy’s head respectively (Fig. 1 and Fig. S1).

### Data acquisition

Readings of the dummy sensors were recorded with a Data Acquisition system (DAQ)—NI USB-6211, National Instruments, Austin, Texas, U.S.A. The trigger signal of the motion capture system was recorded in the sensor data for synchronization. Sensor data was sampled at a frequency of 5 kHz in order to capture the steep peaks in the linear accelerations accurately. The maximum sampling frequency of the motion capture system was only 1 kHz, but this proved sufficient for the purposes of this experiment. Motion data were up-sampled using a low-pass interpolating filter to match the sensor data^59^.

### Data analysis

MathWorks MATLAB (Version R2020b) was used for all calculations, data filtering and statistical analysis. Pearson’s correlation coefficients were determined in IBM SPSS Statistics 25 to check for correlations between participants’ age, weight and height, and the measured and calculated kinematic variables from the cycle containing greatest tangential vertex acceleration by each participant.

### Calculations

The shaking motion occurred mainly in the sagittal plane (x–z-plane), making accelerations in y-direction negligible with respect to accelerations in the x- and z-direction (Fig. S2). Therefore, further analysis was reduced from three to two dimensions to simplify the data processing.

The angle θ (rad) between the z-axis of the dummy’s head and the z-axis of the inertial reference frame was calculated using the positions of the reflective markers. The angular velocity ω (rad/s) was then calculated by differentiation of the angle θ to time and the angular acceleration α (rad/s^2^) by differentiation of the angular velocity ω to time. The maximum angular velocity ω_max_ and maximum angular acceleration α_max_ were obtained from these calculated values of ω and α.

Maximum magnitudes of the resultant torso-, COG- and vertex accelerations (Fig. 1), a_tor-max_ (m/s^2^), a_cog-max_ (m/s^2^), a_ver-max_ (m/s^2^) respectively, were extracted from the sensor data. The vertex sensor x-direction data were differentiated to determine the maximum tangential velocity of the dummy head vertex v_vx-max_ (m/s) with respect to the N frame.

**Figure 6:**
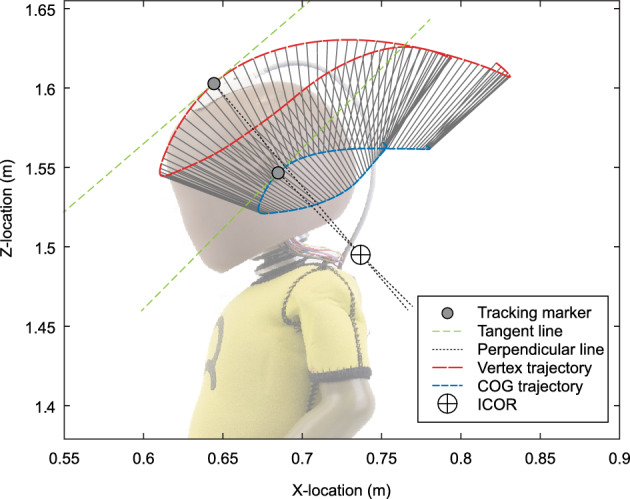
Visual representation of the 3-step calculation of the instantaneous center of rotation (ICOR). Each tangent line is tangential to the marker’s trajectory. The intersection point of the lines perpendicular to the tangent lines is the ICOR; corresponding to a single calculation-step in a single typical shake cycle. The depicted trajectories show a full single typical shake cycle. The solid lines between the vertex and center of gravity (COG) trajectories indicate corresponding datapoints from the same time-step.

The location of the instantaneous center of rotation (ICOR) was calculated for each sample point over the entire duration of the shake cycle (Figs. 2b and 6) with the highest vertex tangential inertial acceleration peak for each participant. To this purpose, at each time step:

1. The tangent lines to the vertex trajectory and the COG trajectory were calculated,
2. For each tangent line a perpendicular line was calculated that projected towards the center of rotation,
3. The location of the ICOR was defined as the intersection point of the two lines perpendicular to the trajectories (Fig. 6). The instantaneous radius of curvature (IROC) was defined as the absolute distance between the ICOR and COG of the dummy’s head.

### Sensitivity analysis

Measurement errors with a normal distribution were simulated and introduced to the experimental data of one randomly selected participant to assess the robustness of the results. The following inaccuracies of the equipment were considered in the sensitivity analysis:

Accelerometer sensitivity of 0.15 g.

1. Motion capture standard deviation of 1.26 mm (2 SD’s were considered for the 95% confidence interval),
2. Trigger delay error of 1 sample (i.e. 0.001 s).

The modeling results (see Supplementary Table S1) showed to be robust to all of these inaccuracies in the experimental setup.

### Data filtering

A Butterworth 12th-order low-pass zero-phase-lag digital IIR filter with a cutoff frequency of 500 Hz was applied to remove noise-frequencies beyond the accelerometer bandwidth and thereby enable the algorithm to calculate the actual acceleration peaks instead of detecting noise peaks. The recorded optical marker trajectories were smoothed to enable fitting tangential lines to these curves. Noise in the motion data would result in errors in the tangent lines and consequently in the calculated location of the rotation center. A power spectral density analysis of the motion data revealed that the signal power beyond 10 Hz was less than − 25 dB. Therefore, the motion data were smoothed using the same Butterworth filter as for the accelerometer data, but with a cutoff frequency of 10 Hz.

### Comparing fixed- and moving-ICOR models accuracies

An overview of existing IHI-ST computational models^12^ was used to identify studies incorporating head dynamics or head kinematics. The kinematics of any point on a shaken head can be defined by a set of equations of motion (EOM)^60^. These were derived for the vertex and COG for each of the found computational models and explained below. All found sagittal plane studies^20–22,35–40^ were fixed-ICOR models and were categorized according to the applied loading directions (Supplementary Tables S2 and S3) and their rotation center definitions.

To improve and complete the replication of head kinematics in IHI-ST in the sagittal plane, we propose to use the “moving-ICOR model”. The Moving-ICOR equations of motion (Eq. 1) contain the complete set of variables necessary to describe the head kinematics including the spatiotemporal variation of the head’s ICOR. Hence, only with this model, the effects of variations in the ICOR location o’ (Fig. 1) can be investigated.

Equations of motion for moving-ICOR models.

1 $$\left\{ {\begin{array}{*{20}c} {\overline{a}_{v} = \overline{\ddot{r}}_{{oo^{\prime}}} + \overline{\alpha } \times \overline{r}_{{o^{\prime}v}} + \overline{\omega } \times \left( {\overline{\omega } \times \overline{r}_{{o^{\prime}v}} } \right) + \overline{\ddot{r}}_{{o^{\prime}v}} + 2\overline{\omega } \times \overline{{\dot{r}}}_{{o^{\prime}v}} + \overline{g}} \\ {\overline{a}_{c} = \overline{\ddot{r}}_{{oo^{\prime}}} + \overline{\alpha } \times \overline{r}_{{o^{\prime}c}} + \overline{\omega } \times \left( {\overline{\omega } \times \overline{r}_{{o^{\prime}c}} } \right) + \overline{\ddot{r}}_{{o^{\prime}c}} + 2\overline{\omega } \times \overline{{\dot{r}}}_{{o^{\prime}c}} + \overline{g}} \\ \end{array} .} \right.$$

With ${\overline{a} }_{v}$ inertial acceleration of the vertex (m/s^2^), ${\overline{a} }_{c}$ inertial acceleration of the COG (m/s^2^), ${\overline{\ddot{r}} }_{o{o}{\prime}}$ inertial acceleration of origin *o’* (m/s^2^), ${\overline{r} }_{{o}{\prime}v}$ position vector from the origin *o’* to the vertex (m), ${\overline{r} }_{{o}{\prime}c}$ position vector from the origin *o’* to the COG (m), ${\overline{\dot{r}} }_{{o}{\prime}v}$ velocity of the vertex relative to origin *o’* (m/s), ${\overline{\dot{r}} }_{{o}{\prime}c}$ velocity of the COG relative to origin *o’* (m/s), ${\overline{\ddot{r}} }_{{o}{\prime}v}$ acceleration of the vertex relative to origin *o’* (m/s^2^), ${\overline{\ddot{r}} }_{{o}{\prime}c}$ acceleration of the COG relative to origin *o’* (m/s^2^), $\overline{\omega }$ angular velocity of the head (rad/s), $\overline{\alpha }$ angular acceleration of the head (rad/s^2^), $\overline{g }$ gravitational acceleration (m/s^2^).

The EOM of fixed-ICOR models are in fact all simplified versions of the EOM of the moving-ICOR model, where in some of these existing models either translation or rotation of the head is neglected, in all but one gravity is neglected, and in all the moving ICOR of the head is neglected. Hence, in each fixed-ICOR model some of the variables in the moving-ICOR model are disregarded or simplified: e.g. ω = 0 and α = 0 in case of translation only, or r_oc_ = constant, ṙ_oc_ = 0 and r̈_oc_ = 0 in case of a fixed rotation center, and *g* = 0 in case gravity was not applied. Thus, the EOM for each fixed-ICOR model category was derived simply by filling in the EOM of the moving-ICOR model, using the specific simplifications of each category:

#### CAT I—Rotation only

Morison^20^, Roth et al.^21^ and Raul et al.^38^ incorporated only rotation in their simulation, around the point o’ (Fig. 1) which was kept fixed over time (Eq. 2). The load application point o’ (Fig. 1) varied per study; Morison^20^ used the base of the neck (**subcategory I-A**), whereas Roth et al.^21^ and Raul et al.^38^ applied the load to the C5-C6 junction of the cervical spine (**subcategory I-B**) (Supplementary Fig. S3). In this special case, the origin o’ was located at the base of the neck or the C5-C6 junction and the z’ axis was aligned with the vertex and COG (Fig. 1). Gravity was neglected in all CAT-I models.

Equations of motion for CAT-I models.

2 $$\left\{\begin{array}{c}{\overline{a} }_{v}=\overline{\alpha } \cdot {\overline{r} }_{{o}{\prime}v}+\overline{\omega }\times \left(\overline{\omega } \cdot {\overline{r} }_{{o}{\prime}v}\right)\\ {\overline{a} }_{c}=\overline{\alpha } \cdot {\overline{r} }_{{o}{\prime}c}+\overline{\omega }\times \left(\overline{\omega } \cdot {\overline{r} }_{{o}{\prime}c}\right)\end{array}.\right.$$

With ${\overline{a} }_{v}$ inertial acceleration of the vertex (m/s^2^), ${\overline{a} }_{c}$ inertial acceleration of the COG (m/s^2^), ${\overline{r} }_{{o}{\prime}v}$ distance from origin o’ to vertex (m), ${\overline{r} }_{{o}{\prime}c}$ distance from origin o’ to COG (m), $\overline{\omega }$ angular velocity of the head (rad/s), $\overline{\alpha }$ angular acceleration of the head (rad/s^2^).

#### CAT II—Translation only

Cheng et al.^35,37^ and Batterbee et al.^36^ incorporated only translation of the head in their simulations, and only in the skull’s horizontal x-direction (Eq. 3). In their studies, the torso acceleration magnitude from a physical model study^30^ was used as inertial acceleration of the head. The load application point may be disregarded; per definition, every point on a purely translating rigid body has the same acceleration. Gravity was neglected in all CAT-II models.

Equations of motion for CAT-II models.

3 $$\left\{\begin{array}{c}{\overline{a} }_{v}={\overline{\ddot{r}} }_{o{o}{\prime}}\\ {\overline{a} }_{c}={\overline{\ddot{r}} }_{o{o}{\prime}}\end{array}\right..$$

With ${\overline{a} }_{v}$ inertial acceleration of the vertex (m/s^2^), ${\overline{a} }_{c}$ inertial acceleration of the COG (m/s^2^), ${\overline{\ddot{r}} }_{o{o}^{\mathrm{^{\prime}}}}$ inertial acceleration of origin o’ (m/s^2^); in this case in the torso.

#### CAT III—Combined rotation and translation

Load application points differed among the studies that incorporated combined rotation and translation (CAT III). Therefore, category III models were sub-classified per load application point; i.e. the base of the neck (CAT III-A) or the base of the skull (CAT III-B). Hans et al.^22^, Couper and Albermani^39^ and Batterbee et al.^40^ incorporated combined rotation and translation in their simulations. Only Hans et al.^22^ applied gravity to the model, hence the addition of gravity in the EOM, and for the other studies gravity was set to zero (Eq. 4). The load application point o’ (Fig. 1) varied per study; Hans et al.^22^ used the base of the neck (**subcategory III-A**), whereas Couper and Albermani^39^ and Batterbee et al.^40^ applied the load to the base of the brainstem—approximately the base of the skull—(**subcategory III-B**) (Supplementary Fig. S3). All these studies used the torso acceleration vector as inertial linear acceleration of the head’s load application point o’ (Fig. 1), and assumed that the torso did not rotate with respect to the inertial reference frame *N*; the angle δ between the torso and the z-axis, and its time derivatives, thus were disregarded (Fig. 1). Therefore, torso translational accelerations that were measured with respect to the torso reference frame *F* must be mapped to the skull reference frame *B* using Eq. (5). The other variables in Eq. (4) were already measured or calculated with respect to the *B*-frame. The subcategories III-A and III-B correspond to our mathematical model, but with the vertex and COG aligned with the origin o’ and the z’ axis, as in Fig. S3.

Equations of motion for CAT-III models.

4 $$\left\{ {\begin{array}{*{20}c} {\overline{a}_{v} = \overline{\ddot{r}}_{{oo^{\prime}}} + \overline{\alpha } \cdot \overline{r}_{{o^{\prime}v}} + \overline{\omega } \times \left( {\overline{\omega } \cdot \overline{r}_{{o^{\prime}v}} } \right) + \overline{g}} \\ {\overline{a}_{c} = \overline{\ddot{r}}_{{oo^{\prime}}} + \overline{\alpha } \cdot \overline{r}_{{o^{\prime}c}} + \overline{\omega } \times \left( {\overline{\omega } \cdot \overline{r}_{{o^{\prime}c}} } \right) + \overline{g}} \\ \end{array} .} \right.$$

With ${\overline{a} }_{v}$ inertial acceleration of the vertex (m/s^2^), ${\overline{a} }_{c}$ inertial acceleration of the COG (m/s^2^), ${\overline{\ddot{r}} }_{o{o}{\prime}}$ inertial acceleration of origin o’ (m/s^2^); i.e. torso acceleration ${\overline{a} }_{t}$, ${\overline{r} }_{{o}{\prime}c}$ distance from origin o’ to COG (m), ${\overline{r} }_{{o}{\prime}v}$ distance from origin o’ to vertex (m), $\overline{\omega }$ angular velocity of the head (rad/s), $\overline{\alpha }$ angular acceleration of the head (rad/s^2^), $\overline{g }$ gravitational acceleration (m/s^2^).

Equation for mapping torso accelerations (that were measured with respect to the torso reference frame F) to the skull reference frame B.

5 $${}^{B}{\overline{a} }_{t}= \left[\begin{array}{ccc}\mathrm{cos}\theta & 0& -\mathrm{sin}\theta \\ 0& 1& 0\\ \mathrm{sin}\theta & 0& \mathrm{cos}\theta \end{array}\right]{}_{ }{}^{F}{\overline{a} }_{t}.$$

With ${}^{B}{\overline{a} }_{t}$ torso acceleration expressed in the skull reference frame *B* (m/s^2^), ${}^{F}{\overline{a} }_{t}$ torso acceleration expressed in the torso reference frame *F* (m/s^2^), $\theta$ angle between the skull reference frame *B* and the z-axis of the inertial reference frame *N* (rad).

After deriving the EOM, the accelerations of the head’s COG and vertex were calculated by feeding the EOM of the proposed moving-ICOR model and of each category of fixed-ICOR models with the kinematic values obtained from the prior experiment. All three models were identically fed with each participant’s data from the prior experiment. In all calculations the dimensions shown in Fig. S3 were used. Finally, the resulting calculated head COG and vertex accelerations were compared to those measured in the same reference dataset that was used as the input for the calculations.

The absolute root-mean-square error (RMSE) between the calculated and measured vertex acceleration was determined over the selected maximum vertex acceleration shake cycle as a quantitative measure of the accuracy of each model. Next, these were averaged over all participants to compare between model categories. The residual accelerations over time (i.e. the difference between the actual reference values and the calculated values of the model) were used to visually assess the accuracy of each model within a shake cycle at all relevant moments.

### Supplementary Information

Supplementary Information.

## Data Availability

The dataset generated and analyzed during the current study and the code used for the analysis are available in the 4TU Centre for Research Data Repository (10.4121/19388672)^47^.

## Abbreviations

IHI-ST: Inflicted head injuries by shaking trauma in infants
FEM: Finite elements model
RBM: Rigid body model
ICOR: Instantaneous center of rotation
IROC: Instantaneous radius of curvature
COG: Center of gravity
EOM: Equations of motion

## References

[1] Fanconi M, Lips U (2010). Shaken baby syndrome in Switzerland: Results of a prospective follow-up study, 2002–2007. Eur. J. Pediatr..

[2] Ellingson KD, Leventhal JM, Weiss HB (2008). Using hospital discharge data to track inflicted traumatic brain injury. Am. J. Prev. Med..

[3] Talvik I (2006). Inflicted traumatic brain injury (ITBI) or shaken baby syndrome (SBS) in Estonia. Acta Paediatr..

[4] Debelle GD, Maguire S, Watts P, Hernandez RN, Kemp AM (2018). Abusive head trauma and the triad: A critique on behalf of RCPCH of 'Traumatic shaking: The role of the triad in medical investigations of suspected traumatic shaking'. Arch. Dis. Child..

[5] Vinchon M, Noizet O, Defoort-Dhellemmes S, Soto-Ares G, Dhellemmes P (2002). Infantile subdural hematomas due to traffic accidents. Pediatr. Neurosurg..

[6] Elinder G (2018). Traumatic shaking: The role of the triad in medical investigations of suspected traumatic shaking. Acta Paediatr. Int. J. Paediatr..

[7] Laghmari M (2014). Birth-related retinal hemorrhages in the newborn: Incidence and relationship with maternal, obstetric and neonatal factors. Prospective study of 2031 cases. J. Fr. Ophtalmol..

[8] Bilo RAC (2018). The Swedish agency for health technology-report about traumatic shaking: Much ado about nothing?. Forensic Sci. Med. Pathol..

[9] Squier W (2008). Shaken baby syndrome: The quest for evidence. Dev. Med. Child Neurol..

[10] Cory CZ, Jones MD (2003). Can shaking alone cause fatal brain injury? A biomechanical assessment of the Duhaime shaken baby syndrome model. Med. Sci. Law.

[11] Lintern TO (2017). Probabilistic description of infant head kinematics in abusive head trauma. Comput. Methods Biomech. Biomed. Eng..

[12] van Zandwijk JP, Vester MEM, Bilo RA, van Rijn RR, Loeve AJ (2019). Modeling of inflicted head injury by shaking trauma in children: what can we learn?: Part II: A systematic review of mathematical and physical models. Forensic Sci. Med. Pathol..

[13] Duhaime AC (1987). The shaken baby syndrome. A clinical, pathological, and biomechanical study. J. Neurosurg..

[14] Prange MT, Coats B, Duhaime AC, Margulies SS (2003). Anthropomorphic simulations of falls, shakes, and inflicted impacts in infants. J. Neurosurg..

[15] Lloyd J, Willey E, Galaznik J, Lee W, Luttner S (2011). Biomechanical evaluation of head kinematics during infant shaking versus pediatric activities of daily living. J. Forensic Biomech..

[16] Tomlinson RA, Taylor ZA (2015). Photoelastic materials and methods for tissue biomechanics applications. Opt. Eng..

[17] Koizumi, T., Tsujiuchi, N., Hara, K., Miyazaki, Y. Dynamic response and damage estimation of infant brain for vibration. In *Proceedings of the 31st international modal analysis conference on structural dynamics. Special topics in structural dynamics*, **6,** 11–18 (2013).

[18] Miyazaki Y (2015). The mechanism of shaken baby syndrome based on the visualization of intracranial brain motion. Jpn. J. Neurosurg..

[19] Jenny CA, Bertocci G, Fukuda T, Rangarajan N, Shams T (2017). Biomechanical response of the infant head to shaking: An experimental investigation. J. Neurotrauma.

[20] Morison CN (2002). The Dynamics of Shaken Baby Syndrome.

[21] Roth S, Raul JS, Ludes B, Willinger R (2007). Finite element analysis of impact and shaking inflicted to a child. Int. J. Legal Med..

[22] Hans SA, Bawab SY, Woodhouse ML (2009). A finite element infant eye model to investigate retinal forces in shaken baby syndrome. Graefes Arch. Clin. Exp. Ophthalmol..

[23] Rangarajan N (2009). Finite element model of ocular injury in abusive head trauma. J. AAPOS.

[24] Yoshida M, Yamazaki J, Mizunuma H (2014). A finite element analysis of the retinal hemorrhages accompanied by shaken baby syndrome/abusive head trauma. J. Biomech..

[25] Nadarasa J, Deck C, Meyer F, Raul JS, Willinger R (2015). Infant eye finite element model to investigate retinal hemorrhages after fall and shaking events. Comput. Methods Biomech. Biomed. Eng..

[26] Bandak FA (2005). Shaken baby syndrome: A biomechanics analysis of injury mechanisms. Forensic Sci. Int..

[27] Schiks LAH, Dankelman J, Loeve AJ (2020). Thresholds for the assessment of inflicted head injury by shaking trauma in infants: a systematic review. Forensic Sci. Int..

[28] Cirovic S, Marco F, Goodwin R, Zimarev D (2012). Shaken Mannequin experiments: Head motion pattern and its potential effect on blood pressure. J. Forensic Biomech..

[29] Jenny, C., Shams, T., Rangarajan, N. & Fukuda, T. Development of a biofidelic 2.5 kg infant dummy and its application to assessing infant head trauma during violent shaking. In *30th International Workgroup on Human Subject Biomechanical Research*, 192–141.

[30] Cheng J (2008). Simulation via Computational and Physical Modelling.

[31] Wolfson DR, McNally DS, Clifford MJ, Vloeberghs M (2005). Rigid-body modelling of shaken baby syndrome. Proc. Inst. Mech. Eng. [H].

[32] Bondy M, Altenhof W, Chen X, Snowdon A, Vrkljan B (2014). Development of a finite element/multi-body model of a newborn infant for restraint analysis and design. Comput. Methods Biomech. Biomed. Eng..

[33] Jones MD, Martin PS, Williams JM, Kemp AM, Theobald P (2015). Development of a computational biomechanical infant model for the investigation of infant head injury by shaking. Med. Sci. Law.

[34] Lintern TO (2015). Head kinematics during shaking associated with abusive head trauma. J. Biomech..

[35] Cheng, J. *et al.* Shaken baby syndrome: A structural dynamics perspective. In *23rd International Conference on Noise and Vibration Engineering 2008, ISMA 2008* **4,** 2003–2014 (2008).

[36] Batterbee, D., Sims, N. & Rowson, J. Finite element modelling of shaken baby syndrome: A frequency response approach. In *Conference Proceedings of the Society for Experimental Mechanics Series* (2009).

[37] Cheng J, Howard IC, Rennison M (2010). Study of an infant brain subjected to periodic motion via a custom experimental apparatus design and finite element modelling. J. Biomech..

[38] Raul JS, Roth S, Ludes B, Willinger R (2008). Influence of the benign enlargement of the subarachnoid space on the bridging veins strain during a shaking event: A finite element study. Int. J. Legal Med..

[39] Couper Z, Albermani F (2010). Mechanical response of infant brain to manually inflicted shaking. Proc. Inst. Mech. Eng. [H].

[40] Batterbee DC, Sims ND, Becker W, Worden K, Rowson J (2011). Computational model of an infant brain subjected to periodic motion simplified modelling and bayesian sensitivity analysis. Proc. Inst. Mech. Eng. [H].

[41] King, A., Yang, K., Zhang, L. & Hardy, W. Is head injury caused by linear or angular acceleration? In *IRCOBI Conference.*

[42] Strich S (1961). Shearing of nerve fibres as a cause of brain damage due to head injury. A pathological study of twenty cases. Lancet.

[43] Holbourn AHS (1943). Mechanics of head injuries. Lancet.

[44] Unterharnscheidt FJ (1971). Translational versus rotational acceleration-animal experiments with measured input. SAE Tech. Paper.

[45] Gennarelli TA, Thibault LE, Ommaya AK (1972). Pathophysiologic responses to rotational and translational accelerations of the head. SAE Tech. Paper.

[46] Gennarelli, T. A., Thibault, L. E. & Ommaya, A. K. Comparison of translational and rotational head motions in experimental cerebral concussion. In *15th Stapp Car Crash Conference*, 797–803.

[47] Schiks, L. A. H., Dankelman, J. & Loeve, A. J. Dataset: *Source data of journal article “Inflicted Head Injury by Shaking Trauma in Infants: potential effect of spatiotemporal variation of the rotation center”.* Repository: 4TU.ResearchData. doi:10.4121/19388672 (2021).

[48] Goldstein B, Kelly MM, Bruton D, Cox C (1993). Inflicted versus accidental head injury in critically injured children. Crit. Care Med..

[49] Ricci L, Giantris A, Merriam Ph, Hodge S, Doyle T (2003). Abusive head trauma in Maine infants: Medical, child protective, and law enforcement analysis. Child Abuse Negl..

[50] Davidsson J, Angeria M, Risling M (2009). Injury threshold for sagittal plane rotational induced diffuse axonal injuries. Proc. Int. Res. Council Biomech. Inj. Conf..

[51] Huang HM, Lee MC, Chiu WT, Chen CT, Lee SY (1999). Three-dimensional finite element analysis of subdural hematoma. J. Trauma Inj. Infect. Crit. Care.

[52] Meany DF (1991). Biomechanics of Acute Subdural Hematoma in the Subhuman Primate and Man.

[53] Lee MC, Melvin JW, Ueno K (1987). Finite element analysis of traumatic subdural hematoma. SAE Tech. Paper.

[54] Raghupathi R, Mehr MF, Helfaer MA, Margulies SS (2004). Traumatic axonal injury is exacerbated following repetitive closed head injury in the neonatal pig. J. Neurotrauma.

[55] Couper ZS, Albermani F (2015). Infant brain subjected to oscillatory loading. Aust. J. Mech. Eng..

[56] Couper Z, Albermani F (2008). Infant brain subjected to oscillatory loading: Material differentiation, properties, and interface conditions. Biomech. Model. Mechanobiol..

[57] Twisk, D. Anthropometric data of children for the development of dummies—TNO report 75061275-B (TNO, 1994).

[58] Stray-Pedersen A, Strisland F, Rognum TO, Schiks LAH, Loeve AJ (2021). Violent infant surrogate shaking: Continuous high-magnitude centripetal force and abrupt shift in tangential acceleration may explain high risk of subdural hemorrhage. Neurotrauma Rep..

[59] IEEE Acoustics Speech & Signal Processing Society (1979). Programs for Digital Signal Processing.

[60] Greenwood DT (2003). Advanced Dynamics.

[61] Couper, Z. & Albermani, F. G. Biomechanics of shaken baby syndrome: Physical testing and numerical modeling. In *18th Australasian Conference on the Mechanics of Structures and Materials.*

