# Supplementary material for: Inflicted head-injury by shaking-trauma in infants: the importance of spatiotemporal variations of the head’s rotation center
Source: Sci Rep. 2023 Sep 14;13:15226. doi: 10.1038/s41598-023-42373-x (PMC10502057; doi:10.1038/s41598-023-42373-x)
Supplement: Supplementary file 1 — Supplementary Information. [file 41598_2023_42373_MOESM1_ESM.docx]

Title: Inflicted Head-Injury by Shaking-Trauma in Infants: the importance of spatiotemporal variations of the rotation center

**Authors:** L. A. H. Schiks^1^, J. Dankelman^1^, A. J. Loeve^1,^^2^*

**Affiliations**

^1^Department of Biomechanical Engineering, Faculty of Mechanical, Maritime and Materials Engineering, Delft University of Technology; Delft, the Netherlands.

^2^Co van Ledden Hulsebosch Center for Forensic Science and Medicine, Amsterdam, the Netherlands.

*Corresponding author. Email: A.J.Loeve@tudelft.nl

**This file includes the following supplementary items:**

Figures S1 to S3 and Tables S1 to S5


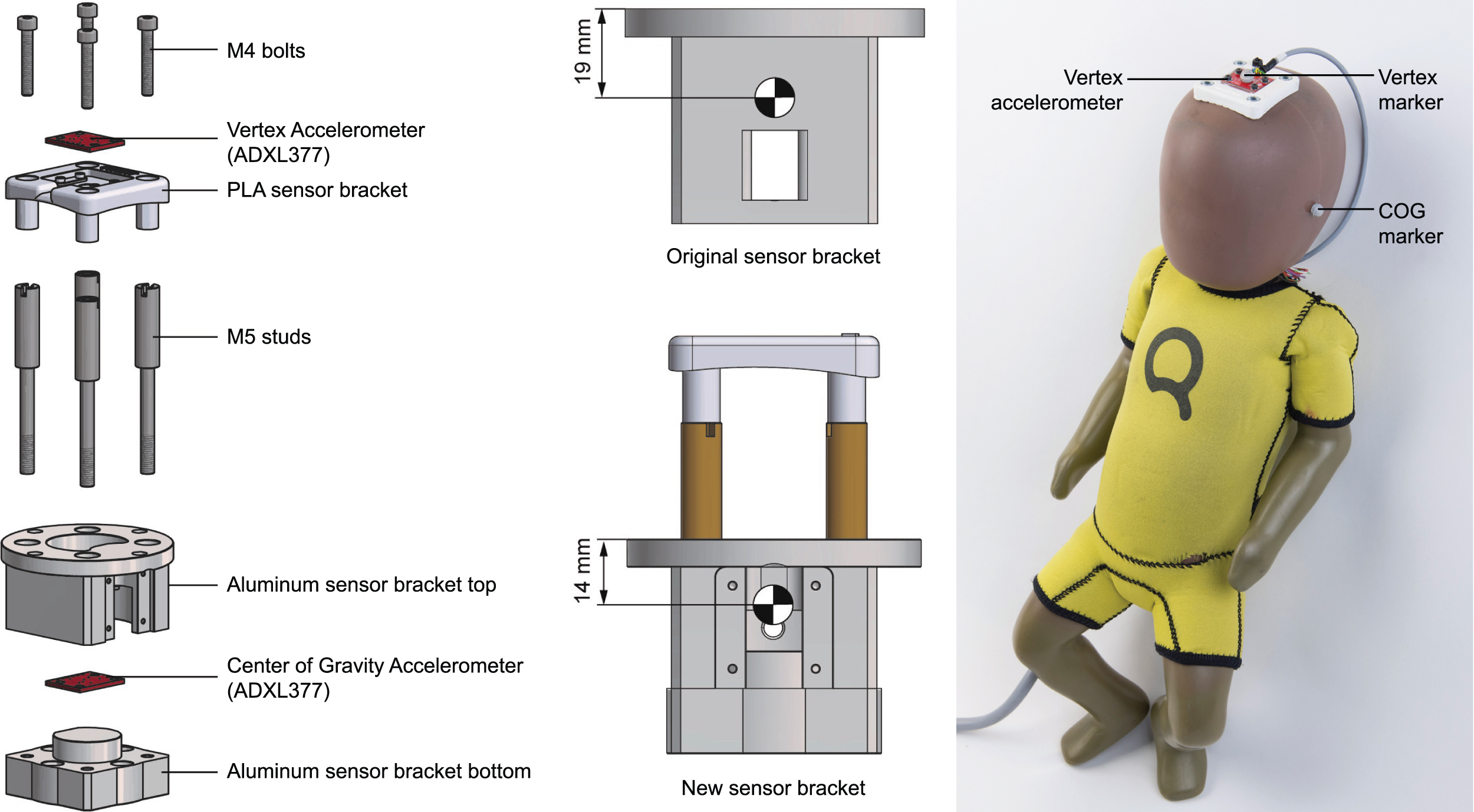


**Supplementary Fig. S1:** Parts of the new sensor bracket (left panel). Center of gravity of the original (center panel, top) and new (center panel, bottom) sensor bracket, and (right panel) full assembly of the modified Q0-dummy. COG: Center of gravity.


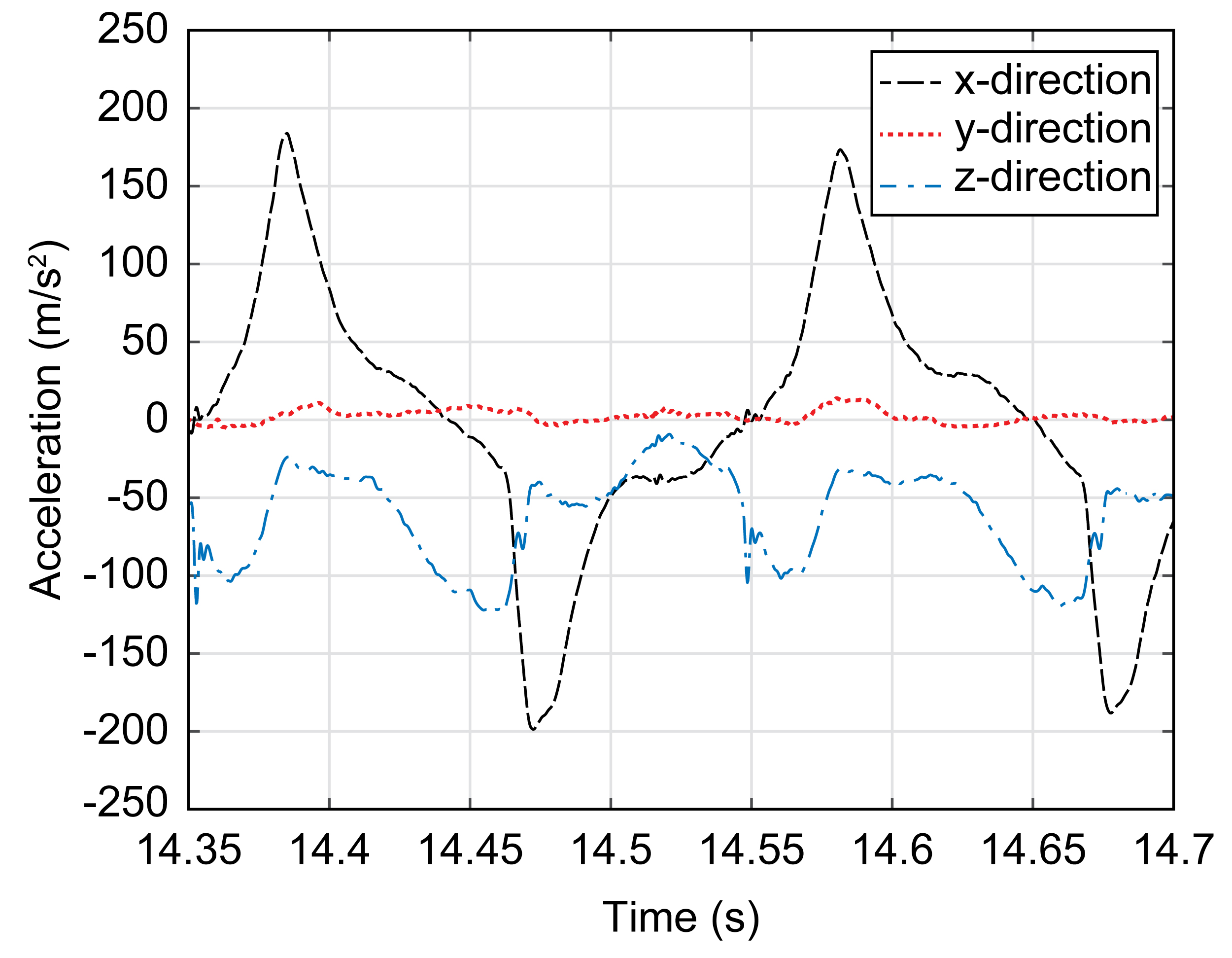


**Supplementary Fig. S2:** Typical vertex accelerations of the dummy’s head during violent shaking.


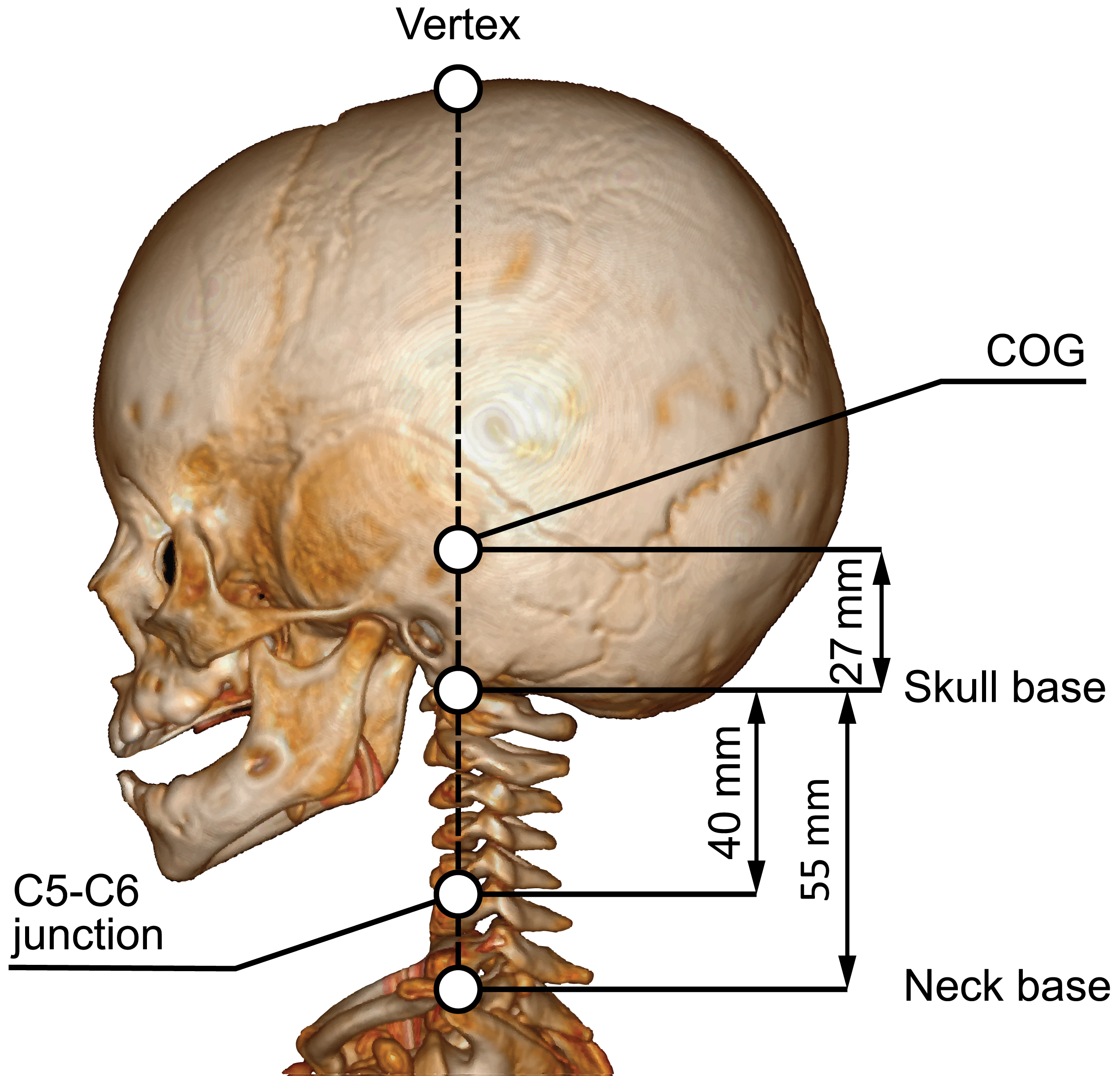


**Supplementary Fig. S3:** Locations of fixed rotation centers used in existing models. 3D CT-scan of a 6-week old infant, the neck length of 55 mm corresponds with the Q0-dummy.

|  | *a_vx_* | *a_vx-acc/MC_** | *a_vx-trig_*** | *a_vz_* | *a_vz-acc/MC_** | *a_vz-trig_*** |
| --- | --- | --- | --- | --- | --- | --- |
| **CAT I-A** | 35.6 | 35.6 | 37.3 | 27.2 | 27.2 | 28.1 |
| **CAT I-B** | 45.4 | 45.4 | 46.8 | 31.1 | 31.1 | 31.9 |
| **CAT II** | 97.3 | 97.3 | 97.3 | N/A | N/A | N/A |
| **CAT III-A** | 30.4 | 30.4 | 33.6 | 21.7 | 21.7 | 22.0 |
| **CAT III-B** | 25.7 | 25.7 | 28.6 | 35.9 | 35.9 | 35.6 |
| **Moving-ICOR model** | 8.9 | 8.9 | 9.3 | 3.1 | 3.1 | 3.8 |

**with simulated inaccuracies in the accelerometer- and motion-capture data*

***with simulated inaccuracy in the trigger delay*

**Supplementary Table S1:** Mean absolute root-mean-square error (RMSE) of calculated vertex acceleration components a_vx_ and a_vz_ of the different models compared to RMSE values incorporating simulated experimental inaccuracies. Only calculated for randomly selected participant 1.

| **Loading direction** | **Model category** |
| --- | --- |
| Rotation around y-axis, rotation center at neck base | CAT I-A |
| Rotation around y-axis, rotation center at C5-C6 junction | CAT I-B |
| Translation in x-direction | CAT II |
| Combined rotation around y-axis and translation in x- and z-direction, rotation center at neck base | CAT III-A |
| Combined rotation around y-axis and translation in x-direction, rotation center at the skull base | CAT III-B |

**Supplementary Table S2:** Categories assigned to head kinematics models used in existing IHI-ST FEM studies.

| **Study** | **Purpose** | **Loading type (and reference if available)** | **Loading direction*** | **Load application point** | **Model category** |
| --- | --- | --- | --- | --- | --- |
| Morison [*20*] | Examine bridging vein strain in a shaking scenario | Head rotation combined with torso acceleration | Rotation in sagittal plane | Rotation center at the base of the neck | I-A |
| Roth et al. [*21*] | Compare intracerebral mechanical response for impact and shaking | Head rotation [*14*] | Rotation in sagittal plane | Rotation center at C5-C6 junction | I-B |
| Cheng et al. [*35*] | Effect of anterior fontanelle in IHI-ST | Torso acceleration [*30*] | Translation in B-frame x-direction | N/A | II |
| Raul et al. [*38*] | Examine influence of the benign enlargement of the subarachnoid space on child head injury | Head rotation [*14*] | Rotation in sagittal plane | Rotation center at C5-C6 junction | I-B |
| Hans et al. [*22*] | Compare retinal forces for impact and shaking | Head rotation combined with torso acceleration [*20*] | Rotation in sagittal plane and translation of rotation center in N-frame x- and z- directions | Rotation center at the base of the neck | III-A |
| Batterbee et al. [*36*] | Development of simplified FEM model and influence of anterior fontanelle | Not clear. Based on magnitude; probably torso acceleration | Translation in B-frame x-direction | N/A | II |
| Cheng et al. [*37*] | Examine influence of anterior fontanelle and brain-skull interface on intracranial brain movement | Torso acceleration [*30*] | Translation in B-frame x-direction | N/A | II |
| Couper and Albermani [*39*] | Examine the loading–injury relationship of infant shaking and the related deterministic parameters | Head rotation combined with torso acceleration [*13, 14, 31, 61*] | Rotation in sagittal plane and translation of rotation center in N-frame x-direction | Rotation center at the base of the brainstem (approx. skull base) | III-B |
| Batterbee et al. [*40*] | Uncertainty analysis of a finite element model of an infant’s head subject to shaking | Head rotation combined with torso acceleration | Rotation in sagittal plane and translation of rotation center in N-frame x-direction | Rotation center at the base of the brainstem (approx. skull base) | III-B |

***Loading directions corresponding to the reference frames in Figure 1.**

**Supplementary Table S3:** Existing studies were each assigned a model category (see Table S2 for definitions) according to their loading type, loading direction and load application point.

|  | *a_tor-max_* | *a_cog-max_* | *a_ver-max_* | *_max_* | α*_max_* | *v_vx-max_* | *f_shake_* |
| --- | --- | --- | --- | --- | --- | --- | --- |
| **Age** | -.154 (.427) | -.271 (.156) | -.241 (.209) | -.202 (.293) | -.263 (.168) | -.188 (.329) | .081 (.677) |
| **Height** | .205 (.285) | .116 (.549) | .149 (.439) | .202 (.294) | .194 (.314) | .176 (.362) | .289 (.128) |
| **Weight** | .220 (.250) | .046 (.812) | .126 (.516) | .264 (.166) | .164 (.395) | .173 (.370) | .151 (.434) |

**Supplementary Table S4:** Correlation coefficient matrix of the study variables (Pearson’s r (sig.)). None of the correlations were significant (p>0.05).

|  | *a_tor-max_ (m/s^2^)* | *a_cog-max_ (m/s^2^)* | *a_ver-max_ (m/s^2^)* | *_max_ (rad/s)* | α*_max_ (rad/s^2^)* | *v_vx-max_* *(m/s)* | *f_shake_ (Hz)* |
| --- | --- | --- | --- | --- | --- | --- | --- |
| **Maximum** | 257 | 276 | 606 | 66 | 6,010 | 7.0 | 6.2 |
| **Mean** | 123 | 149 | 300 | 43 | 2,977 | 4.3 | 4.5 |
| **SD** | 49 | 48 | 113 | 11 | 1,017 | 1.2 | 0.8 |

**Supplementary Table S5:** Peak magnitudes of the shaking variables across all participants.
